# Hospitalization outcomes in people living with HIV on Dolutegravir-based regimen in Mwanza, Tanzania: a comparative cohort

**DOI:** 10.1186/s12981-025-00706-y

**Published:** 2025-02-03

**Authors:** Godfrey A. Kisigo, Eric Barongo, Benson Issarow, Cody Cichowitz, Bahati Wajanga, Samuel Kalluvya, Robert N. Peck

**Affiliations:** 1 Department of Internal Medicine, Weill Bugando School of Medicine, Mwanza, Tanzania; 2 Department of Infectious Disease Epidemiology, London School of Hygiene and Tropical Medicine, London, UK; 3 Mwanza Intervention Trials Unit, National Institute for Medical Research, Mwanza, Tanzania; 4 Division of Cardiology, University of California San Francisco, San Francisco, USA; 5 Center for Global Health, Weill Cornell Medicine, New York, NY USA

**Keywords:** Tanzania, HIV, Hospitalization, Mortality, Dolutegravir, HIV multi-morbidity

## Abstract

**Introduction:**

Hospitalized people living with HIV (PLWH) experienced extremely high mortality rates in the first year after an index hospitalization in the pre-Dolutegravir (DTG) era. We conducted a multi-center study in Mwanza, Tanzania to (1) describe causes of hospitalization for PLWH on DTG; (2) determine in-hospital and 3-month post-hospital mortality; (3) examine factors associated with overall mortality; and (4) determine changes in trends and predictors of mortality pre- and post-DTG era.

**Methods:**

Between August 2020 and February 2021, hospitalized PLWH on dolutegravir-based antiretroviral therapy were enrolled and followed for three months after hospitalization. The primary outcome was mortality within 3-months of hospitalization. Cox regression analysis was used to calculate hazard ratios to identify predictors of mortality.

**Results:**

Of the 154 enrolled patients, the median [interquartile range] age was 42 [33–50] years and 57% were female. Suspected immune reconstitution inflammatory syndrome (IRIS) and antiretroviral therapy (ART) non-adherence leading to an HIV-associated admission were common. The overall all-cause mortality was 42%. Male sex, using DTG-based regimen for < 3 months, diagnosis of suspected IRIS, diagnosis of ART side effect, advanced WHO clinical stage, CD4 count < 200 cells/mm^3^, hemoglobin level 7–11.9 gm/dl and hemoglobin level < 7 gm/dl were all independent risk factors for death.

**Conclusion:**

In conclusion, the mortality rate of hospitalized PLWH in Africa remains high in the DTG era. Clinical trials are urgently needed to test novel interventions for improving survival in this high-risk group. In the meantime, hospital clinicians should be aware of the very high mortality among PLWH with IRIS and those with ART side effect to ensure that all possible diagnostic and therapeutic options are explored.

**Supplementary Information:**

The online version contains supplementary material available at 10.1186/s12981-025-00706-y.

## Background

In 2019, the World Health Organization (WHO) produced updated guidelines for the treatment of HIV [1]. Tanzania subsequently adopted a dolutegravir (DTG)-based regimen as first-line antiretroviral therapy (ART) in the same year [2]. This regimen includes a combination of DTG (an integrase strand-transfer inhibitor) and two nucleoside reverse-transcriptase inhibitors, namely tenofovir and lamivudine. The DTG-based regimen has been shown to achieve rapid viral suppression and immunological recovery [3]. Furthermore, DTG is reported to have a high genetic barrier to resistance and is available at a low cost [4]. These attributes made DTG-based ART the standard, first-line regimen for almost all people living with HIV (PLWH) in Africa.

During the pre-DTG era, we and others documented how hospitalized PLWH experience extremely high mortality rates in the first year after an index hospitalization. We reported that nearly a third (31%) of adults living with HIV died within 3-month of hospital discharge in Tanzania [5]. Others have reported a similarly high rate of post-hospital mortality in South Africa, Malawi, Uganda, and Botswana [6–9]. There is a lack of data on hospitalization and health outcomes among PLWH in the DTG era.

Therefore, we conducted a multi-center study at three government hospitals in Mwanza, Tanzania to (1) describe causes of hospitalization for PLWH on DTG, (2) determine in-hospital and 3-month post-hospital mortality, (3) examine factors associated with overall mortality, and (4) determine changes in trends and predictors of mortality pre- and post-DTG era. We determined that 130 people living with HIV would be required to estimate the expected 3-month mortality of 31.4% with a precision of ± 8% at a 95% confidence level. The expected 3-month mortality was based on mortality reported in our previous cohort [5].

## Methods

Between August 2020 and February 2021, we recruited 154 hospitalized PLWH on DTG-based ART at the time of hospital admission and followed them for 3 months after discharge. After providing written informed consent, participants completed a standardized questionnaire that included questions about demographic and HIV-related information. Hospital record review was performed to extract clinical diagnoses and treatments. A trained physician performed clinical examination. Follow-up surveys were conducted monthly via phone calls according to protocols established in prior studies [5, 10]. This study was approved by the Joint Ethics Committee of the Catholic University of Health and Allied Science and Bugando Medical Center.

The primary outcome was all-cause mortality within 3-months of hospitalization. The vitality status of study participants was determined during the monthly phone calls. If the participant was not available by phone, the alternative contact was called to determine the participant's health status. The cause of death was determined by reviewing medical records if death occurred in-hospital or by verbal autopsy for those who died at home. The primary diagnosis leading to hospitalization was classified by two investigators independently after reviewing the patient’s hospital record or interviewing relatives. Since we were interested in the impact of a DTG-based regimen on HIV treatment, we specified diagnoses for all causes of hospitalization that are HIV-related conditions and ART treatment outcomes. The remaining causes of hospitalization were classified as due to other infectious or non-infectious diseases.

Baseline demographic and clinical characteristics were summarized by median and interquartile range for continuous variables and frequency and percentage for categorical variables. Cox regression analysis was used to calculate hazard ratios to identify predictors of 3 months post hospitalization mortality. We used a time-dependent covariate to test the assumption of proportional hazards. A 2- P sided value of less than 0.05 was regarded as statistically significant. Analysis was conducted in Stata (v17, StataCorp LLC, College Station, TX).

## Results

During the study period, 260 adults with HIV were hospitalized at study sites. Of these 260, 87 (33%) were newly diagnosed and ART naïve. Among 173 PLWH who had previously been prescribed ART, 162 (93%) were on DTG-based ART, 8 (5%) were on PI-based second-line ART, and 3 (2%) were on NNRTI-based ART.

The 162 patients were on DTG-based ART and were screened for enrollment. Of these, 5 did not have telephone access and 3 did not provide consent to participate. The remaining 154 (95%) were prescribed a DTG-based ART and were enrolled. Baseline characteristics are described in Table 1. Of the 154 enrolled patients, the median [interquartile range] age was 42 [33–50] years and 88 (57%) were female. The median duration of ART was 373 [72–455] days and 75 (49%) had CD4 count of < 200 cell/mm^3^.

**Table 1:** Baseline demographic and clinical characteristics by the vitality status at 3 months post hospitalization (N = 154)

Predictor	All (N = 154)	Alive (N = 89)	Dead (N = 65)
Sex			
Females	88 (57%)	58 (65%)	30 (46%)
Males	66 (43%)	31 (35%)	35 (54%)
Age (years), median [IQR]	42 [33–50]		
≥ 50	36 (23%)	22 (25%)	14 (22%)
34–49	83 (54%)	43 (48%)	40 (61%)
18–33	35 (23%)	24 (27%)	11 (17%)
Level of Education			
No formal Education	32 (21%)	22 (25%)	10 (15%)
Primary school education	86 (56%)	48 (54%)	38 (59%)
Secondary /college/University	36 (23%)	19 (21%)	17 (26%)
Occupation			
Contractual labor	57 (37%)	34 (38%)	23 (35%)
Farmers	39 (25%)	17 (19%)	22 (34%)
Employed—full time/business owner	31 (20%)	19 (21%)	12 (19%)
Unemployed	27 (18%)	19 (21%)	8 (12%)
Duration on dolutegravir, median [IQR]	373 [72–455]		
> 1 year	81 (52%)	54 (61%)	27 (42%)
≥ 3 months–1 year	29 (19%)	17 (19%)	12 (18%)
< 3 months	44 (29%)	18 (20%)	26 (40%)
Cause of hospitalization			
Other infectious disease	36 (23%)	25 (28%)	11 (17%)
ART side effects	4 (3%)	0 (0%)	4 (6%)
Suspected IRIS	23 (15%)	9 (10%)	14 (22%)
ART non-adherence^a^	22 (14%)	10 (11%)	12 (18%)
Other non-infectious disease	69 (45%)	45 (51%)	24 (37%)
WHO Staging of AIDS			
Stage 1 and 2	63 (41%)	47 (53%)	16 (25%)
Stage 3 and 4	91 (59%)	42 (47%)	49 (75%)
Body mass index (kg/m^2^), median [IQR]	19.1 [17.0–22.2]		
Normal weight	79 (51%)	46 (52%)	33 (51%)
Underweight	61 (40%)	33 (37%)	28 (43%)
Overweight	14 (9%)	10 (11%)	4 (6%)
Hemoglobin level (gm/dl), median [IQR]	9.7 [7.1–11.9]		
≥ 12	37 (24%)	27 (30%)	10 (15%)
7–11.9	83 (54%)	45 (51%)	38 (59%)
< 7	34 (22%)	17 (19%)	17 (26%)
CD4 (per 1 unit of CD4), median [IQR]	205 [73–395]		
≥ 350	49 (32%)	36 (40%)	13 (20%)
200–350	30 (19%)	21 (24%)	9 (14%)
< 200	75 (49%)	32 (36%)	43 (66%)

IQR, Interquartile Range

^a^Being off ART for more than 7 days

Suspected IRIS (15%, n = 23) and ART non-adherence leading to an HIV-associated admission (14%, n = 22) were common. Only 4 patients (3%) were diagnosed with ART side effects, which were all clinically diagnosed as suspected tenofovir nephropathy. The leading causes of hospitalization were other non-communicable diseases (45%, n = 69), followed by other infectious diseases (23%, n = 36). Supplementary Table 1 describes specific diagnoses for other non-communicable and infectious diseases.

The median hospital stay was 5 [3–9] days. A total of 36 /154 (23%) participants died during the hospital stay. Among 118 participants who survived hospital discharge, 29/118 (25%) were reported dead at the end of the 3-month follow-up. The overall all-cause mortality was 42% (n = 65). Of the 118 PLWH discharged home with an indication for preventive therapy, only 26% (n = 31) and 5% (n = 6) of the participants were on Cotrimoxazole and Isoniazid preventive therapies, respectively.

**Fig. 1:**
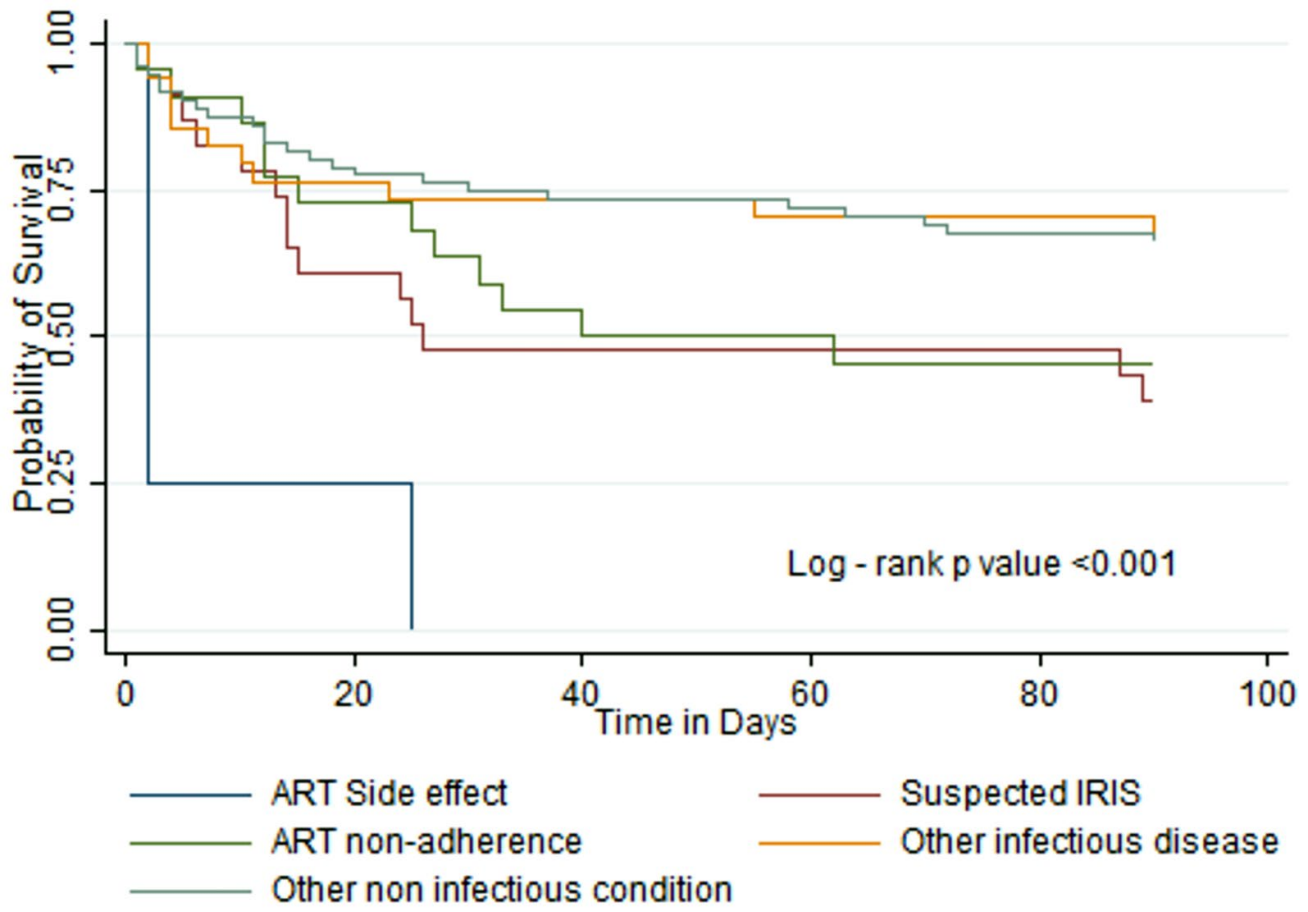
displays Kaplan Meier survival curves for the 5 diagnostic groups (p < 0.001)

Table 2 reports predictors of overall mortality in hospitalized PLWH. In the final multivariable Cox model, the factors independently associated with mortality were: male sex, using DTG-based regimen for < 3 months, diagnosis of suspected IRIS, diagnosis of ART side effect, advanced WHO clinical stage, CD4 count < 200 cells/mm3, hemoglobin level 7–11.9 gm/dl and hemoglobin level < 7 gm/dl (see Fig. 1). Mortality predictors before the DTG-based regimen are presented in Supplementary Table 2.

**Table 2:** Predictors of mortality within 3 months

Predictor	Crude analysis	p-value	Adjusted analysis	p-value
	HR [95% CI]		aHR* [95% CI]	
Sex				
Females	1.00		1.00	
Males	1.72 [1.06–2.81]	0.029	1.75 [1.03–2.96]	0.037
Age (years)				
≥ 50	1.00			
34–49	1.38 [0.75–2.53]	0.302		
18–33	0.78 [0.35–1.72]	0.544		
Level of education				
No formal Education	1.00			
Primary school education	1.50 [0.75–3.01]	0.255		
Secondary /college/University	1.67 [0.76–3.65]	0.199		
Duration on dolutegravir				
> 1 year	1.00		1.00	
≥ 3 months–1 year	1.27 [0.64–2.50]	0.493	1.50 [0.75–3.01]	0.253
< 3 months	2.12 [1.23–3.64]	0.006	2.26 [1.29–3.96]	0.004
Cause of hospitalization				
Other infectious disease	1.00		1.00	
ART side effects	10.0 [3.11–32.44]	< 0.001	24.45 [6.26–95.42]	** < 0.001**
Suspected IRIS	2.20 [0.99–4.85]	0.051	2.33 [1.02–5.30]	0.044
ART non-adherence^a^	1.81 [0.80–4.12]	0.154	1.90 [0.81–4.47]	0.142
Other non-infectious disease	1.02 [0.50–2.08]	0.959	1.02 [0.49–2.10]	0.956
WHO Staging of AIDS				
Stage 1 and 2	1.00		1.00	
Stage 3 and 4	2.58 [1.47–4.54]	0.001	2.70 [1.51–4.84]	0.001
Body mass index (kg/m^2^)				
Normal weight	1.00			
Underweight	1.10 [0.67–1.83]	0.701		
Overweight	0.61 [0.22–1.72]	0.351		
Hemoglobin level (gm/dl)				
≥ 12	1.00		1.00	
7–11.9	1.87 [0.93–3.74]	0.080	2.96 [1.43–6.14]	0.003
< 7	2.23 [1.02–4.87]	0.045	3.29 [1.46–7.41]	0.004
CD4 (per 1 unit of CD4)				
≥ 350	1.00		1.00	
200–350	1.14 [0.49–2.67]	0.757	1.15 [0.48–2.72]	0.753
< 200	2.75 [1.48–5.13]	0.001	3.05 [1.61–5.77]	0.001

^*^Adjusted for age, sex, and occupation

^a^Being off ART for more than 7 days

## Discussion

In this study, we re-examined the outcomes of PLWH hospitalized in the Mwanza Region of Tanzania during the DTG era compared to a study conducted before DTG availability. The in-hospital and 3-month post-hospital mortality were similar to what we reported from the same hospitals five years ago (23% vs. 27% (χ^2^ (1, N = 154) = 1.2, p = 0.2776), 25% vs. 31% (χ^2^ (1, N = 118) = 2.5, p = 0.1124), respectively) before adopting the DTG-based regimen [5]. DTG-based regimens are safe in advanced HIV disease but may not reduce mortality or non-adherence [11–13]. This is particularly relevant among hospitalized PLWH who are likely to have advanced illness with multiple opportunistic infections [14–16]. As such, hospital and post-hospital care improvements remain critical in the DTG era, as recently recommended by the WHO [17].

Predictors of mortality were generally similar in this population receiving DTG-based ART compared to our prior cohort conducted before DTG availability. Male sex, recent initiation of ART, CD4 count < 200, and anemia were independent risk factors for death in both cohorts. Interestingly, in this new cohort, admission with ART side effects (i.e., suspected tenofovir nephropathy) and IRIS were also important predictors of mortality. Whether this is directly related to DTG or not cannot be determined from this study. Clinicians, though, should be aware of these risk factors and provide tailored clinical care to improve health outcomes. In addition, since non-communicable diseases were the leading causes of hospitalization in PLWH taking DTG, there is need of a holistic approach in providing care to PLWH which addresses both infectious and non-communicable disease [18–20].

This study has several limitations that must be considered. First, we relied on hospital diagnoses made by clinicians in resource-limited hospitals. Thorough history taking, physical examination, and standard tests were performed to establish the cause of hospitalization. diagnoses were aligned with the International Classification of Diseases 10th Revision (ICD-10) codes. Secondly, HIV viral load testing was not performed at the time of hospitalization since this is not part of the standard of care in Tanzania. The rapid suppression of viral load is an advantage of DTG-based regimen compared to non-integrase inhibitor-based regimens. The similar rates of post-discharge mortality observed in the pre-DTG and DTG era may be confounded by possible differences in baseline viral loads at the time of recruitment.

## Conclusion

In conclusion, the mortality rate of hospitalized PLWH in Africa remains high in the DTG era. Clinical trials are urgently needed to test novel interventions for improving survival in this high-risk group. In the meantime, hospital clinicians should be aware of the very high mortality among PLWH with IRIS and those with ART side effect to ensure that all possible diagnostic and therapeutic options are explored.

## Supplementary Information

Additional file 1.

## Data Availability

The datasets used and/or analyzed during the current study are available from the corresponding author on reasonable request.

## Abbreviations

ART: Antiretroviral therapy
DTG: Dolutegravir
PLWH: People living with HIV
WHO: World Health Organization
