# Supplementary material for: Hospitalization outcomes in people living with HIV on Dolutegravir-based regimen in Mwanza, Tanzania: a comparative cohort
Source: AIDS Res Ther. 2025 Feb 3;22:11. doi: 10.1186/s12981-025-00706-y (PMC11789402; doi:10.1186/s12981-025-00706-y)
Supplement: Supplementary file 1 — Additional file 1. [file 12981_2025_706_MOESM1_ESM.docx]

Supplemental Table 1. Specific Diagnoses for Other infectious diseases and other non-infectious diseases

| Other non-infectious diseases (N=69) | | Other infectious diseases (N=36) | |
| --- | --- | --- | --- |
| Diagnosis | n (%) | Diagnosis | n (%) |
| Abdominal tumor | 1 (1.4%) | Bacterial pneumonia | 7 (19.4) |
| Anemia | 3 (4.3%) | Extra pulmonary tuberculosis | 3 (8.3%) |
| HIV associated Nephropathy | 2 (2.9%) | TB meningitis | 1 (2.8%) |
| Hepatic encephalopathy | 3 (4.3%) | Abdominal wall pyomyositis | 1 (2.8%) |
| Kaposi’s Sarcoma | 3 (4.3%) | Abdominal TB | 1 (2.8%) |
| Non-hodgkin lymphoma | 2 (2.9%) | Bacterial meningitis | 10 (27.8%) |
| Abdominal tumor | 1 (1.4%) | Dysentery | 1 (2.8%) |
| Alcoholic liver disease | 1 (1.4%) | Gastroenteritis | 4 (11.1%) |
| Bladder tumor | 2 (2.9%) | Malaria | 3 (8.3%) |
| Breast mass | 1 (1.4%) | Pneumocystis pneumonia | 1 (2.8%) |
| Cholangiocarcinoma | 1 (1.4%) | Post TB structural | 1 (2.8%) |
| Decompensated congestive heart failure | 2 (2.9%) | Urine tract infection | 2 (5.6%) |
| Deep vein thrombosis | 1 (1.4%) | Vaginal candidiasis | 1 (2.8%) |
| Dysfunctional uterine bleeding | 11 (15.9%) |  |  |
| Epilepsy | 1 (1.4%) |  |  |
| Frontal bone lytic mass | 1 (1.4%) |  |  |
| Guillain barre syndrome | 2 (2.8%) |  |  |
| Hepatocellular carcinoma | 3 (4.2%) |  |  |
| Hepatorenal syndrome | 1 (1.4%) |  |  |
| Hypertensive urgency | 1 (1.4%) |  |  |
| Inguinal hernia | 1 (1.4%) |  |  |
| Intrauterine fetal death | 1 (1.4%) |  |  |
| Ovarian carcinoma | 1 (1.4%) |  |  |
| Ovarian mass | 1 (1.4%) |  |  |
| Peripheral neuropathy | 1 (1.4%) |  |  |
| Psychosis | 2 (2.9%) |  |  |
| Stroke | 4 (5.8%) |  |  |
| Uncontrolled diabetes mellitus | 3 (4.3%) |  |  |
| Upper gastrointestinal bleeding | 8 (11.6%) |  |  |
| Urethral stricture | 1 (1.4%) |  |  |

Supplemental Table 2. Predictors of mortality during pre-DTG era. Predictors are listed by significance of association (Peck et al., 2016).

| Variable | OR | 95% CI | p-value |
| --- | --- | --- | --- |
| **(A) Predictors of in-hospital mortality** |  |  |  |
| Oxygen saturation |  |  |  |
| Linear | 0.89 | 0.84 – 0.94 | < 0.001 |
| Quadratic | 0.99 | 0.99 – 0.99 | 0.003 |
| Systolic hypotension (binary: <90 mm Hg) | 9.02 | 2.77 – 29.38 | < 0.001 |
| Glasgow coma scale (categorical: <13, 13–14, 15) | 0.36 | 0.26 – 0.51 | < 0.001 |
| Proteinuria by urinalysis (binary) | 2.26 | 1.41 – 3.63 | 0.001 |
| History of diabetes | 0.22 | 0.08 – 0.62 | 0.004 |
| Heart rate (beats per minute) | 1.02 | 1.00 – 1.02 | 0.008 |
| Current tobacco smoking | 3.03 | 1.30 – 7.09 | 0.01 |
| Bilateral leg edema | 1.92 | 1.15 – 3.21 | 0.012 |
| Male gender | 1.71 | 1.07 – 2.71 | 0.024 |
| **(B) Predictors of post-hospital mortality** |  |  |  |
| Less than primary level education | 2.44 | 1.60 – 3.72 | 0.001 |
| Proteinuria by urinalysis | 1.51 | 1.17 – 1.94 | 0.001 |
| Systolic blood pressure (mm Hg) | 0.99 | 0.98 – 0.99 | 0.002 |
| Bilateral leg edema | 2.17 | 1.30 – 3.61 | 0.003 |
| HIV | 1.99 | 1.22 – 3.24 | 0.006 |
| Oxygen saturation | 0.96 | 0.93 – 0.99 | 0.013 |

Peck, R. N., Wang, R. J., Mtui, G., Smart, L., Yango, M., Elchaki, R., Wajanga, B., Downs, J. A., Mteta, K., & Fitzgerald, D. W. (2016). Linkage to Primary Care and Survival After Hospital Discharge for HIV-Infected Adults in Tanzania: A Prospective Cohort Study. *JAIDS Journal of Acquired Immune Deficiency Syndromes*, *73*(5), 522–530. https://doi.org/10.1097/QAI.000000000001107
